# From Earth to orbit: How to preserve muscle health in space and bed rest

**DOI:** 10.1113/EP092729

**Published:** 2025-03-26

**Authors:** Antonios Matsakas, Colleen Deane

**Affiliations:** ^1^ Department of Life Sciences Manchester Metropolitan University Manchester UK; ^2^ Human Development and Health, Faculty of Medicine, Southampton General Hospital University of Southampton Southampton UK

**Keywords:** atrophy, bed rest, exercise, skeletal muscle, spaceflight

## 1 CURRENT INSIGHTS INTO MUSCLE ATROPHY

Muscle mass and strength loss during spaceflight and prolonged bed rest are inevitable consequences of disuse. Extensive evidence suggests that muscle atrophy under both conditions is primarily driven by impaired protein synthesis and reduced protein turnover. A recent review published in this Special Issue of *Experimental Physiology* by Bosutti et al. (2025) critically discusses the physiological mechanisms associated with microgravity‐induced loss of muscle mass and function, as well as the latest countermeasures aimed at mitigating its effects (Figure 1 in Bosutti et al., 2025). At the molecular level, dysregulation of the Akt/mechanistic target of rapamycin signaling pathway, which governs protein synthesis, along with activation of multiple proteolytic pathways, such as the ubiquitin–proteasome system and autophagy, play a central role in muscle wasting. Additionally, disruptions in cytoskeletal integrity and extracellular matrix organization lead to spatial disarray of cellular organelles, resulting in mitochondrial and nuclear dysfunction. Such structural changes may also compromise neuromuscular junctions, potentially explaining the disproportionate decline in muscle strength relative to muscle mass, as observed in bed rest studies (Table 2 in Bosutti et al., 2025). Beyond structural impairments, both spaceflight and prolonged bed rest induce significant metabolic disturbances, including substrate inflexibility, mitophagy, lipotoxicity, mito‐nuclear communication deficits and insulin resistance, all of which highlight the urgent need for effective countermeasures against muscle wasting – both on Earth and in orbit.

## 2 EXERCISE, NUTRITIONAL AND NEUROMUSCULAR COUNTERMEASURES

Among the available interventions, exercise remains the most effective strategy for mitigating muscle mass and strength decline in microgravity (Figure 2 in Bosutti et al., 2025). Various regimens, including a combination of aerobic, resistance and vibration exercises, have shown promise in attenuating muscle atrophy. Notably, reactive jumps involving eccentric contractions have emerged as particularly effective, offering both muscular and cardiovascular benefits during prolonged bed rest and spaceflight (Thomasius et al., 2023). However, a key challenge for exercise‐based countermeasures in space remains the size of exercise equipment and the difficulty in achieving high resistance loads (Rittweger et al., 2018). Additionally, the potential benefits of artificial gravity via centrifugation along the major body axis as a means to mitigate muscle loss remains under investigation. Whether such biomechanical interventions can replicate Earth‐like loading conditions to effectively counteract atrophy in space remains an open question. Beyond exercise, nutritional supplementation plays a crucial role in offsetting negative energy balance and maintaining protein turnover. Key nutritional interventions include whey protein and the leucine metabolite β‐hydroxy β‐methylbutyrate, which support muscle maintenance and enhance the anabolic response to exercise (Gao & Chilibeck, 2020; Wilkinson et al., 2013). Another promising approach, particularly for immobilized individuals on Earth, is neuromuscular electrical stimulation, which has demonstrated efficacy in preventing muscle mass loss following prolonged disuse (Hardy et al., 2022; Maffiuletti et al., 2019). However, its effectiveness in space remains to be fully established (Figure 3 in Bosutti et al., 2025).

## 3 FUTURE WIDER IMPLICATIONS: BEYOND SPACEFLIGHT AND BED REST

The future of muscle atrophy countermeasures likely lies in multimodal strategies that integrate exercise, nutrition, biomechanical interventions and post‐recovery rehabilitation. While pharmacological approaches remain largely experimental, they hold potential as synergistic interventions. For instance, selective androgen receptor modulators exhibit anabolic effects similar to testosterone but with fewer side effects, while vitamin D plays a critical role in maintaining muscle function and bone health, particularly in microgravity environments. Importantly, research on countermeasures against muscle atrophy extends beyond space exploration, offering valuable insights into broader clinical and public health contexts. This is because mechanisms underlying muscle loss in astronauts and immobilized patients share striking similarities with those observed in sarcopenia (i.e., the age‐related loss of muscle mass, strength and function), neurodegenerative diseases, muscular dystrophies and critical illness‐induced muscle wasting. Anabolic resistance in ageing (i.e., blunted response to protein intake and exercise) contributes to muscle wasting, reduced mobility and increased morbidity. Countermeasures explored in space research, such as exercise training, protein supplementation and neuromuscular stimulation, could be applied to older adult populations to maintain muscle health and functional independence and vice versa. Similarly, bed rest immobilization due to injury or intensive care treatment is followed by rapid muscle atrophy and strength loss with inevitably impaired long‐term quality of life. The development of targeted countermeasures, including electrical stimulation, artificial gravity and pharmacological interventions, could support rehabilitation strategies for these people and patients. Beyond clinical applications, athletes recovering from sports injuries may also benefit from spaceflight‐inspired countermeasures. Periods of forced inactivity due to fractures, ligament tears or post‐surgical recovery can lead to significant muscle loss. Eccentric exercise, neuromuscular electrical stimulation and nutritional strategies may mitigate atrophy and accelerate rehabilitation. Moreover, understanding metabolic inflexibility and insulin resistance in microgravity has implications for conditions such as type 2 diabetes and metabolic syndrome. Disrupted mito‐nuclear communication and lipid metabolism observed in astronauts mirror metabolic dysfunction seen in obesity and diabetes. Future research may therefore contribute to novel therapeutic approaches for improving glucose regulation, mitochondrial health and metabolic flexibility in populations at risk. Ultimately, the interplay of space physiology and terrestrial medicine provides a unique opportunity to develop holistic, evidence‐based strategies for preserving muscle health across diverse populations. By continuing to explore multimodal countermeasures, researchers can enhance human performance, rehabilitation and ageing interventions, ensuring that both astronauts and patients on Earth benefit from cutting‐edge advancements in muscle biology.

## AUTHOR CONTRIBUTIONS

Both authors have read and approved the final version of this manuscript and agree to be accountable for all aspects of the work in ensuring that questions related to the accuracy or integrity of any part of the work are appropriately investigated and resolved. All persons designated as authors qualify for authorship, and all those who qualify for authorship are listed.

## CONFLICT OF INTEREST

None declared.

## FUNDING INFORMATION

No funding was received for this work.
